# Atypical Distant Metastasis of Breast Malignant Phyllodes Tumors: A Case Report and Literature Review

**DOI:** 10.1155/2017/8963013

**Published:** 2017-10-11

**Authors:** Tiphaine de Foucher, Hélène Roussel, Mikael Hivelin, Léa Rossi, Caroline Cornou, Anne-Sophie Bats, Myriam Deloménie, Fabrice Lécuru, Charlotte Ngô

**Affiliations:** ^1^Department of Breast and Gynecological Surgical Oncology, Hôpital Européen Georges Pompidou, AP-HP, Paris, France; ^2^Department of Pathology, Hôpital Européen Georges Pompidou, AP-HP, Paris, France; ^3^Department of Plastic and Reconstructive Surgery, Hôpital Européen Georges Pompidou, AP-HP, Paris, France; ^4^Paris Descartes University, Sorbonne Paris Cité, Paris, France

## Abstract

Malignant phyllodes tumors (MPT) are rare breast neoplasms. Preoperative diagnosis is often challenging due to the unspecific clinical, radiological, and histological characteristics of the tumor. Dissemination pathways are local with chest wall invasion, regional with lymph nodes metastasis, and distant, hematogenous, mostly to the lungs, bones, and brain. Distant metastasis (DM) can be synchronous or appear months to years after the diagnosis and initial management. The current report describes the case of a 57-year-old woman presenting with a giant/neglected MPT of the breast, with no DM at initial staging, treated by radical modified mastectomy. Motor disorders due to medullar compression by a paravertebral mass appeared at short follow-up, also treated surgically. The patient died from several DM of rapid evolution. To our knowledge, this is the only case described of MPT with metastases to soft tissue causing medullar compression. We present a literature review on unusual metastatic localizations of MPT.

## 1. Introduction

Phyllodes tumors (PT) are fibroepithelial tumors characterized by a double-layered epithelial component arranged in clefts surrounded by an overgrowing mesenchymal component organized in leaf-like structures. Grading between benign, borderline, or malignant depends on histological criteria: stromal cellularity, cellular pleomorphism, mitotic activity, margin appearance, and stromal distribution [1]. The average annual incidence rate of malignant phyllodes tumors (MPT) is 2.1 per 1 million women [2].

Surgery allowing tumor-free margins ≥ 1 cm is the first line of treatment [3]. Axillary lymph node dissection is recommended in case of palpable nodes [4]. Radiotherapy and chemotherapy can be options in case of high metastatic risk or recurrence.

Considering all PT, local recurrence (LR) and distant metastases (DM) rates are estimated around 20% and 3.5%, respectively [5]. Considering MPT, LR occurs in 40% and DM in 27% of the cases, mostly to lungs, bones, brain, and liver [6–9]. The estimated 5-, 10-, and 15-year rates of cause-specific survival for women operated for primary nonmetastatic MPT are 91%, 89%, and 89%, respectively [3].

We report the case of a woman with an atypical metastasis from a MPT with a rapidly fatal outcome. We present a literature review about unusual localizations of MPT metastasis.

## 2. Case Presentation

**Figure 1:**
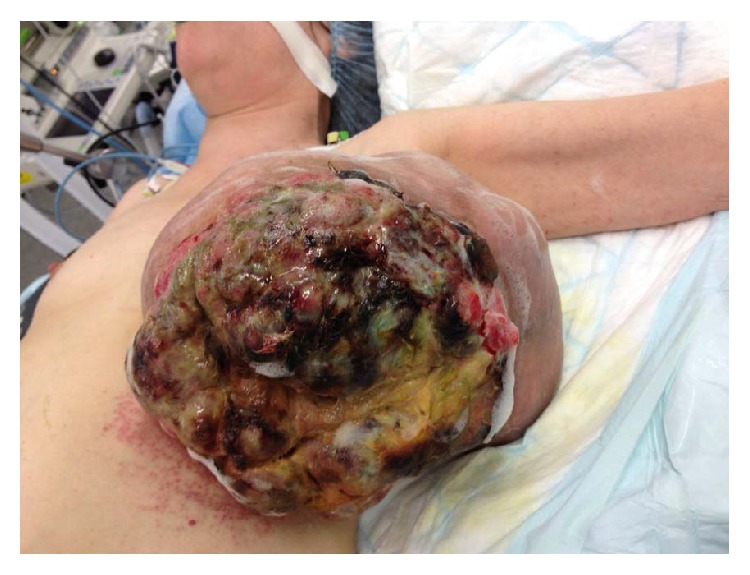
Picture of the left breast mass at diagnosis.

**Figure 2:**
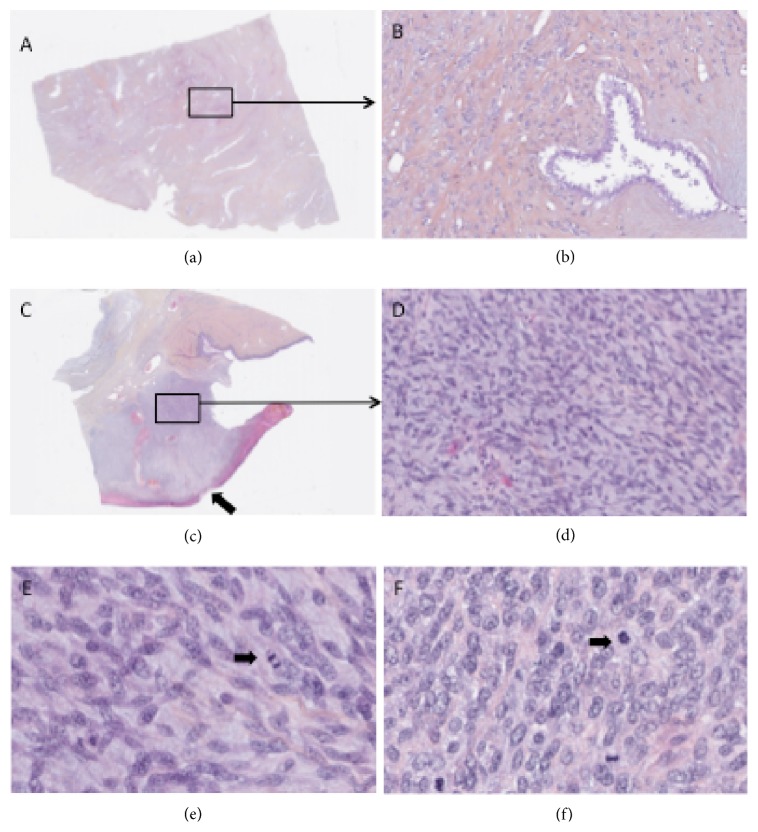
Microscopic aspect of the primary tumor (a, b, c, d, e) and medullar metastasis (f). (a, b) Predominant benign PT component. The stroma is more cellular than a fibroadenoma. Stromal cellularity may be higher in the zone adjacent to epithelium without atypia and mitosis ((a) magnification ×2, (b) magnification ×200), (c, d) malignant PT component observed near the cutaneous ulceration (arrow). We observed a high stromal cellularity with atypia and mitosis without epithelial structure. There was no involvement of the tissue beneath the nipples nor lymphovascular or neural invasion. ((c) magnification ×2, (d) magnification ×200), (e, f) malignant primary (e), and medullar metastasis (f) PT at magnification ×400. We observed a similar histologic pattern: a cellular stromal proliferation with atypia and mitosis (arrows). No epithelial components. All immunostainings were negative (estrogen receptor, progesterone receptor, pankeratin AE1, AE3, and PS100, desmin, CD34, caldesmon, and CD99) except for a focal staining with Smooth Muscle Actin antibody.

A 57-year-old menopausal woman presented herself to our institution with a giant necrotic breast tumor and ipsilateral axillary lymphadenopathy (Figure 1). Radical mastectomy with axillary node dissection and partial pectoral muscle resection was performed. Bulk size was 10,6 × 5,9 × 6,3 inches and it weighted 4.2 kg. The majority of the lesion was composed of a benign PT but a focal area presented a bulging high grade malignant PT with severity criteria: infiltrative borders, high mitotic count, marked stromal overgrowth, and marked stromal cellularity (Figure 2). Axillary lymph nodes were disease-free. Postoperative PET-CT showed no distant metastasis.

Few weeks later, the patient came back with cervical and back pain. She also showed a delirious melancholic episode, successfully treated with neuroleptics.

**Figure 3:**
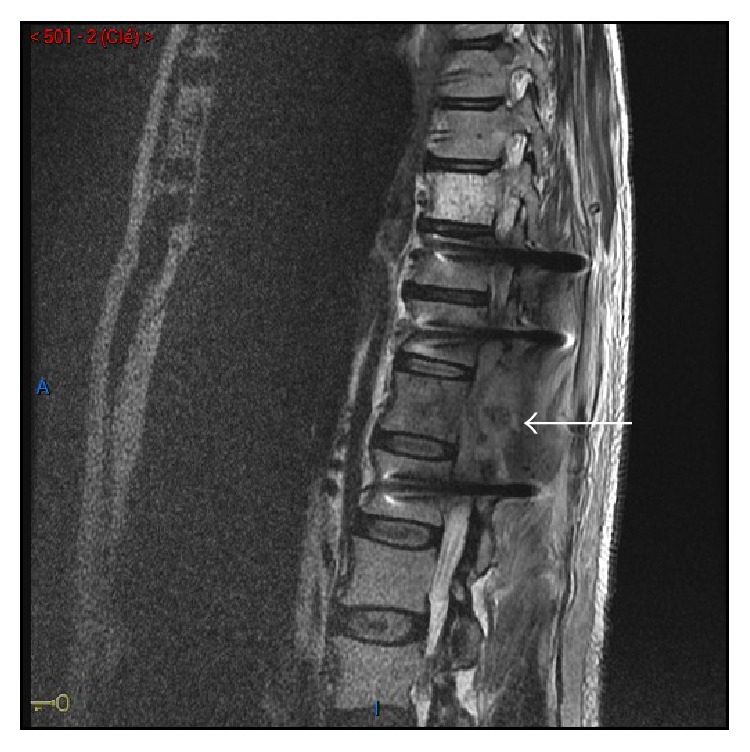
Spinal MRI revealing paravertebral lesions between T11-T12 causing significant mass effect on the conus medullaris.

The spinal MRI revealed several paravertebral lesions: a right paravertebral soft tissue tumor extending from C3 to C5 causing spine displacement and a mass in T11-T12 causing mass effect on the conus medullaris (Figure 3). The occurrence of a cauda equina syndrome indicated an emergency surgery. Histopathological analysis showed a high malignancy tumor proliferation with clusters of spindle shaped cells. The phenotype was unspecific but comparable to the breast tumor (Figure 2).

She then presented multiple and rapidly growing metastasis in soft tissues. Chemotherapy with Adriamycin was initiated but she died rapidly, 4 months after primary diagnosis.

## 3. Discussion and Literature Review

We presented an unusual case of rapidly fatal metastatic evolution of a breast MPT with atypical distant metastasis to paravertebral tissues.

In their retrospective analysis of 295 patients, Mitus et al. found that five-year DFS was 96.9% in patients with benign PT, 83.3% in patients with borderline PT, and 71.7% in patients with malignant PT [7]. 95% of deaths were related to distant metastasis of malignant PT. The mean survival in case of metastasis was 7 months [range 2–17]. These results are consistent with other articles, which show that metastatic PT carries a poor prognosis, with an average survival time of less than 2 years [3, 6, 10].

Several grading systems have been proposed, but the three-tiered system including benign, borderline, and malignant PT is preferred [11]. The grading is based on semiquantitative assessment of infiltrative borders, stromal overgrowth, stromal cellularity, stromal pleomorphism, mitotic count (≥5 mitoses per 10 HPF), and the presence of a malignant heterologous component [9, 12, 13]. In our case, in the primary tumor, the histologic criteria of MPT were a high mitotic count with marked stromal overgrowth, high stromal cellularity, and atypia. No malignant heterologous component was found.

The patient waited for more than 18 months before consulting. She demonstrated a strong denial of the disease and of the treatments that were planned. In addition, this patient was in a situation of socioeconomic deprivation. Observing low-income women, Nonzee et al. studied the reasons of delayed breast cancer screening, follow-up, and treatment [14]. They showed that despite equal access to cancer care-related services, common explanations for nonadherence included limited knowledge about preventive or cancer care resources and denial or fear. Furthermore, it appears that women with locally advanced breast cancer are more likely to suffer from psychiatric comorbidity and more often live alone [15]. In our case, the melancholic episode may have delayed the diagnosis of soft tissue metastases, due to the denial of imaging and medical treatment of the patient. A more effective comanagement of the patient involving both surgeons and psychiatrists might have improved care.

**Figure 4:**
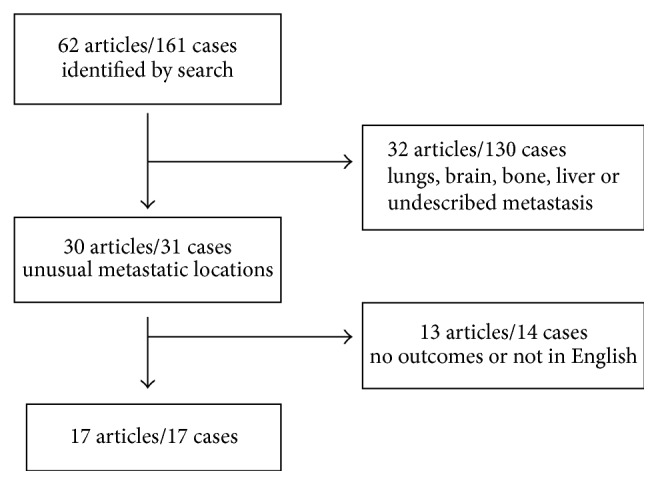
Selection for review of literature: references were obtained from the PubMed database, using the keywords “phyllodes tumor metastasis”.

Selection of review of literature for unusual metastasis is summarized in Figure 4. We finally retrieved 17 articles reporting 17 cases. Three patients suffered of cardiac localizations [16–18]. Four patients had gastrointestinal localizations [19–22]. One patient presented with a borderline PT of the right breast and simultaneous pancreatic tail metastasis [23]. Five patients presented with ENT metastasis [24–28]. One patient presented with a thyroid mass two years after a simple mastectomy for a MPT [29]. One case of left kidney metastasis has been reported [30] and one case of adrenal metastasis [31]. The last patient presented with thoracic vertebra and rib metastasis, as well as a pelvic mass [32]. Cases are summarized in Table 1. Mean DFS was 25 months; mean OS was 49 months. 15 patients were dead at the time of publication because of the disease. This highlights the very poor prognosis of patient with unusual DM of MPT.

In case of initially nonmetastatic MPT with high risk of recurrence, adjuvant therapy including radiotherapy and/or different chemotherapeutic agents (ifosfamide, etoposide, doxorubicin, or cisplatin) can be used, although their role is uncertain [4]. Here the rapid apparition of multiple metastases, despite a negative postoperative PET-CT and free surgical margins, raises the question of systematic postoperative radiotherapy and chemotherapy in case of large MPT. In case of distant metastasis, chemotherapy can be used, as well as postoperative radiation therapy as palliation for pain relief, but with limited efficacy [33]. In our case, chemotherapy was quickly started after metastases diagnosis, but the progression of the disease was so fast that the patient died after only one cycle.

Recently, the potential key role of genomic markers in the characterization of PT has been highlighted. MED12 somatic mutations have been identified as a highly recurrent event in fibroadenomas (FAs) and phyllodes tumors (PTs), with an inverse correlation between the frequency of this mutation and histologic grade [34–36]. Laé et al. identified a limited number of altered signaling pathways associated with this mutation, suggesting the use of these findings as diagnostic and prognostic tools [37]. Focusing on MPT, other authors used molecular profiling to identify overexpressed biomarkers of angiogenesis, EGFR, and immune checkpoints, which points the way toward the use of new targeted therapies [38].

MPT are uncommon breast neoplasm, whose prognosis can be very poor in case of DM. Medullar compression due to soft tissue metastasis is extremely rare. This strengthens the value of an accurate initial diagnosis, so as to enable the identification of high-risk patients. Their management, including monitoring and treatment, is yet to be determined, as the efficiency of treatments used for DM is still low.

## Figures and Tables

**Table 1:** Summary of the review of literature.

Year of publication	Author	Site of distant metastasis	Delay between primary and metastasis (months)	Treatment	OS and DFS (months)
Present case	T. de Foucher	Paravertebral tissue	1	CT	OS = 4
2016	Yoshiba [24]	Left mandible	36	RT	DFS = 18, OS = 42
2016	Shan [32]	Pelvis	53	Surgery and CT	DFS = 16, OS = 72
2016	Choi [19]	Stomach	64	Endoscopy and PPI	DFS = 35, OS = 68
2015	Karczmarek-Borowska [30]	Left kidney	10	Surgery, palliative RT CT	DFS = 10, OS = 17
2015	Yoshidaya [16]	Heart	4	Surgery	DFS = 4, OS = 6
2014	Wei [23]	Pancreas		Surgery and CT	DFS = 39, OS = 41
2014	Sano [25]	Left tonsil	71	Surgery	DFS = 3
2013	Collin [31]	Adrenal gland	96	Surgery	DFS = 12, OS = 108
2012	Bilen [20]	Jejunum	12	Surgery	DFS = 13
2011	Garg [17]	Heart	36	Surgery	DFS = 5, OS = 36
2010	Nakatsu [18]	Heart	108	Surgery	DFS = 108, OS = 111
2010	Morcos [21]	Jejunum	13	Surgery	DFS = 13, OS = 31
2006	Masmoudi [26]	Gingiva	24	Unknown	DFS = 24, OS = 25
2006	Asoglu [22]	Duodenum	72	Surgery	DFS = 24
2003	Deeming [27]	Mandible	108	Palliative RT	DFS = 72, OS = 114
2003	Staton [28]	Mandible	12	Palliative RT	DFS = 12, OS = 13
2002	Giorgadze [29]	Thyroid	24	Surgery	DFS = 24, OS = 48

OS, overall survival; DFS, disease-free survival; CT, chemotherapy; RT, radiotherapy; PPI, proton pump inhibitors.
